# Small Bowel Obstruction Secondary to Sigmoid Diverticulitis With Reactive Enteritis: Diagnosis via CT and Successful Conservative Management

**DOI:** 10.7759/cureus.97058

**Published:** 2025-11-17

**Authors:** Mohammed Babiker, Antonio Gallucci, Ahmed Ahmed

**Affiliations:** 1 Emergency General Surgery, Wirral University Teaching Hospital NHS Foundation Trust, Wirral, GBR; 2 Radiology, Wirral University Teaching Hospital NHS Foundation Trust, Wirral, GBR

**Keywords:** antibiotics therapy, complicated diverticulitis, computed tomography (ct ), conservative management, functional obstruction, reactive enteritis, small bowel obstruction (sbo), water-soluble contrast challenge

## Abstract

Small bowel obstruction (SBO) is a common cause of acute abdominal presentation. While most cases are mechanical, secondary functional obstruction from inflammatory processes is rare. Inflammation of the sigmoid colon can, in some instances, extend to adjacent small bowel loops, resulting in reactive changes and transient obstruction. A man in his 60s presented with abdominal pain, distension, and fever. Contrast-enhanced CT of the abdomen and pelvis demonstrated sigmoid diverticulitis with localized perforation and pericolic inflammation, surrounded by mildly dilated small bowel loops showing subtle mural enhancement, consistent with reactive change. He was managed conservatively with nasogastric decompression, IV fluids, and broad-spectrum antibiotics as per local guidelines. Total parenteral nutrition was commenced after five days of no oral intake to prevent nutritional deterioration. A water-soluble contrast study was performed for diagnostic and therapeutic purposes, confirming bowel continuity and promoting resolution of the obstruction. The patient improved clinically, resumed oral intake, and was discharged after seven days with complete recovery. This case highlights a rare mechanism of SBO secondary to sigmoid diverticulitis, in which reactive inflammatory changes in the adjacent small bowel caused functional narrowing, without evidence of a discrete mechanical transition point. Prompt recognition of reactive enteric changes on CT allows appropriate conservative management. Control of the primary colonic inflammation led to resolution of the reactive changes and restoration of bowel function without surgical intervention.

## Introduction

Small bowel obstruction (SBO) is a frequent surgical emergency, most commonly caused by postoperative adhesions [1]. It represents a major source of hospitalization and morbidity in general surgical practice. While adhesive obstruction predominates, SBO secondary to colonic diverticulitis is exceptionally uncommon.

While colonic diverticulitis is a prevalent condition, with a lifetime risk estimated at 10-25% in Western populations, its complications typically include abscess formation, perforation, fistula, or peritonitis rather than obstruction [2]. SBO secondary to diverticulitis is exceedingly rare, with only sporadic cases described in the literature [3,4].

When present, obstruction is usually caused by extrinsic compression from an inflammatory phlegmon, abscess, or adhesional band formation rather than intrinsic small bowel disease [3]. Reactive inflammatory changes in adjacent small bowel loops may also occur, leading to functional narrowing or dysmotility rather than a true mechanical blockage [4,5].

CT is the diagnostic modality of choice, as it can accurately delineate the transition point, bowel wall changes, and associated pericolic inflammation, thereby distinguishing functional obstruction from mechanical causes [5].

Recognition of this rare mechanism is clinically important, as it supports a nonoperative management approach in stable patients, avoiding unnecessary surgery and its associated morbidity [1,3].

## Case presentation

A man in his 60s presented with a two-day history of suprapubic pain radiating to the left iliac fossa and flank, progressive abdominal distension, and subjective fever (38.9°C at home). He had passed stool the day before but was constipated on admission. He reported streaky hematuria but denied hematochezia or mucus.

He had a history of gout, angina managed with an elective percutaneous coronary intervention to the mid left anterior descending artery, recurrent urinary tract infections, sciatica, and previous spinal surgery. His regular medications included allopurinol for gout, bisoprolol and aspirin for angina, rosuvastatin for hyperlipidemia, gabapentin for neuropathic pain, and tamsulosin for benign prostatic symptoms. He had no history of abdominal surgery or known diverticular disease. He was allergic to plasters. He lived with his family, was independent in activities of daily living, and had minimal alcohol intake with no smoking history.

On examination, he was hemodynamically stable (RR: 19/min, SpO₂: 97% on room air, BP: 140/90 mmHg, HR: 73 bpm, and temperature: 36.7°C). The abdomen was grossly distended, tympanic to percussion, and tender in the hypogastric and left iliac fossa regions, with reduced bowel sounds. An umbilical hernia was reducible, and there were no peritoneal signs. Blood tests demonstrated leukocytosis (WCC 16 × 10⁹/L), markedly raised CRP (340 mg/L), hyponatremia (Na: 129 mmol/L), and acute kidney injury stage [1] (creatinine: 140 µmol/L) with normal hemoglobin (146 g/L) and liver function tests.

**Figure 1:**
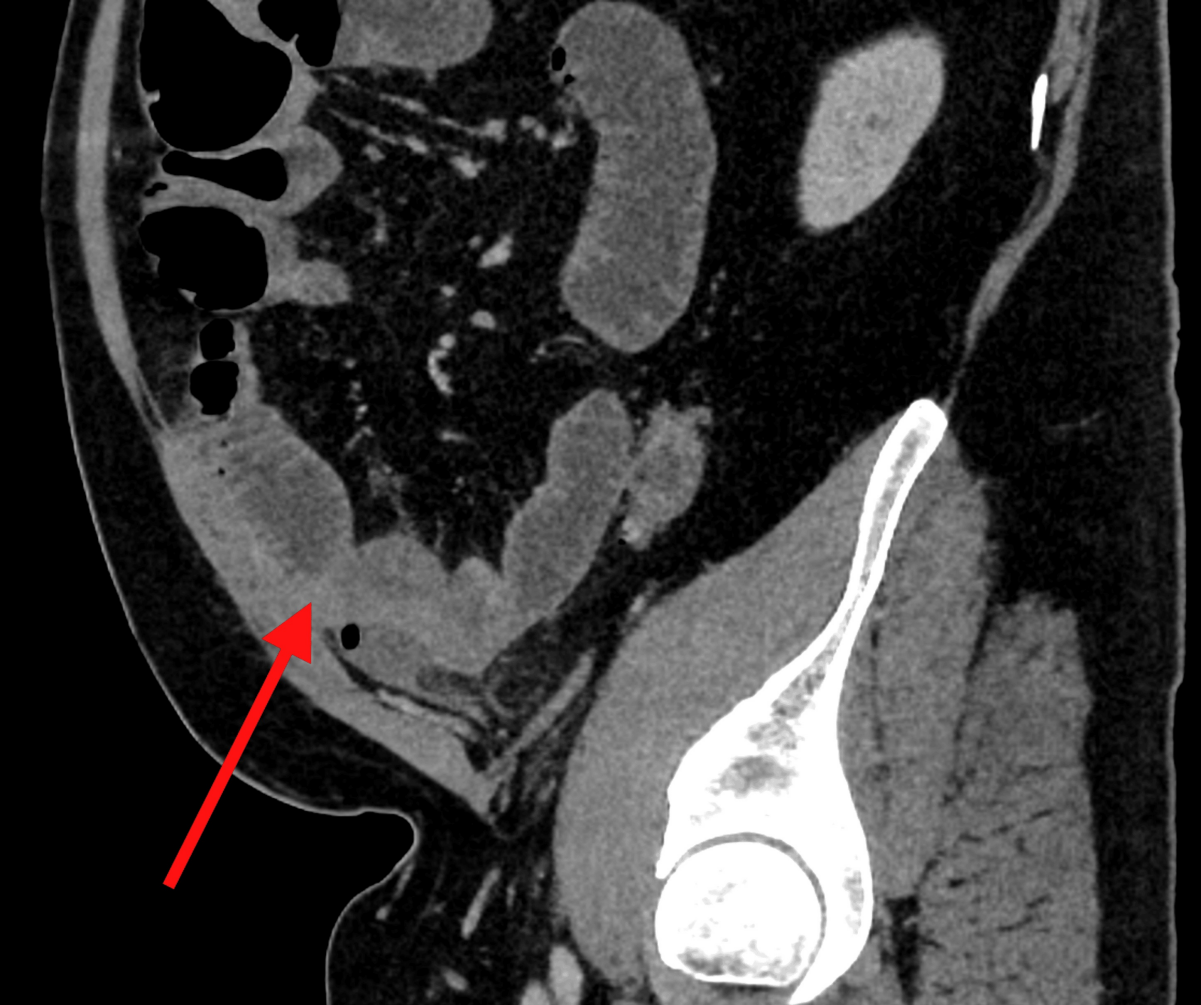
Sagittal view of contrast-enhanced CT of the abdomen The red arrow highlights the caliber change between dilated and collapsed small bowel loops, consistent with the transition point near the inflamed sigmoid colon.

**Figure 2:**
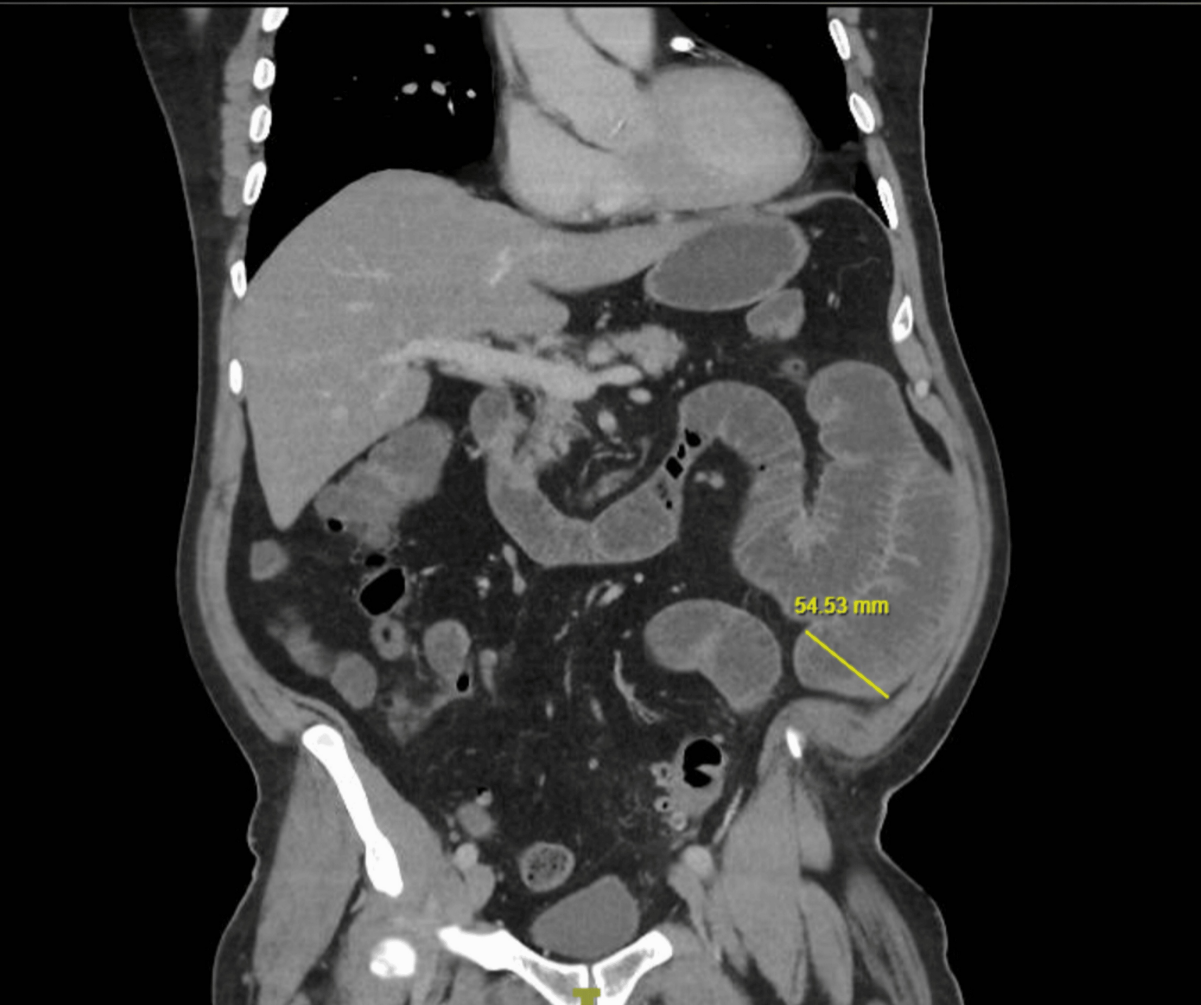
Coronal view of contrast-enhanced CT of the abdomen Dilated small bowel loops (up to 54 mm) with mural thickening and enhancement are seen adjacent to the sigmoid colon, consistent with reactive enteritis.

**Figure 3:**
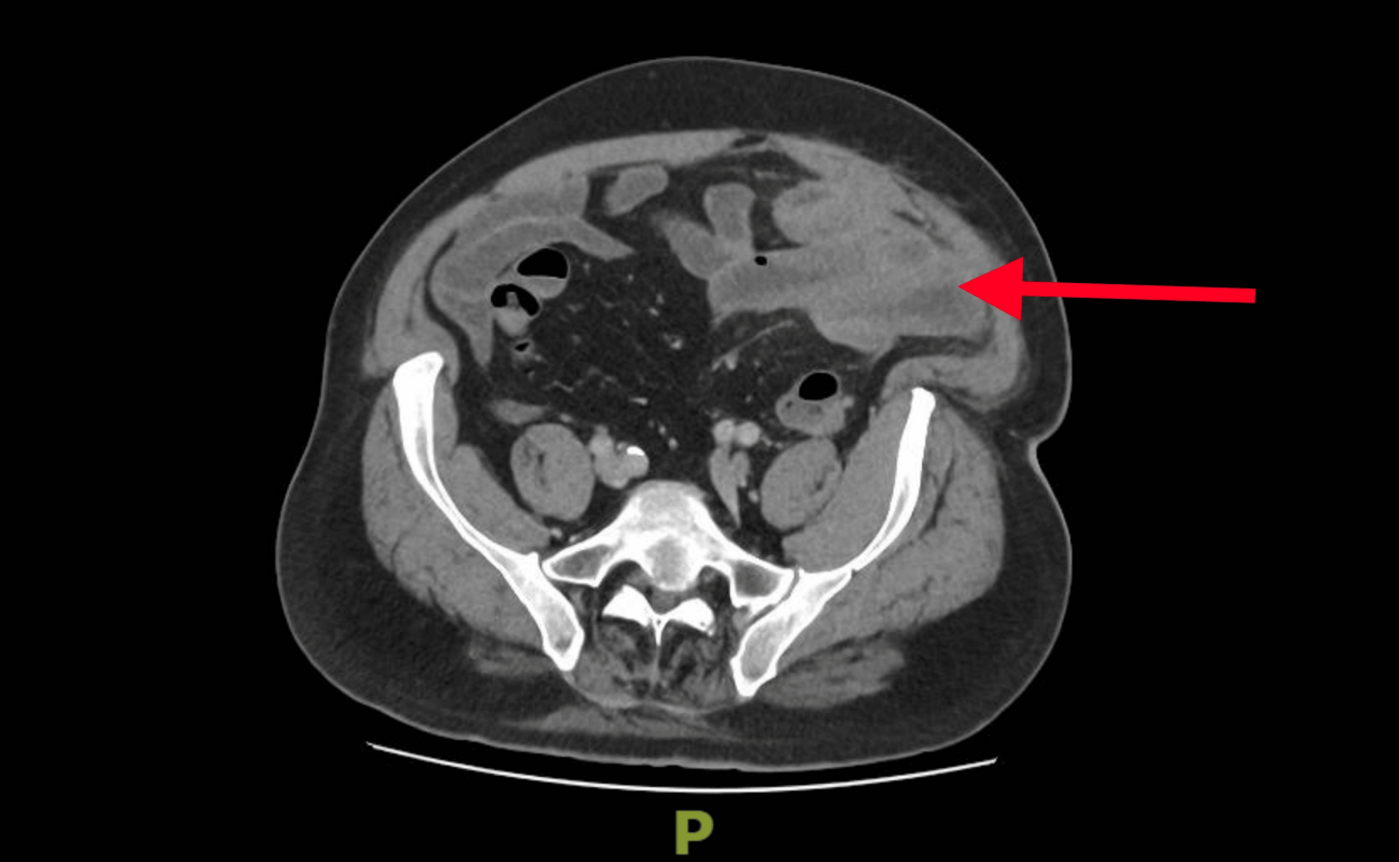
Axial view (superior slice) of contrast-enhanced CT of the pelvis The red arrow highlights adjacent small bowel loops showing mural hyperenhancement and mild edema, representing reactive enteritis secondary to adjacent sigmoid diverticulitis.

**Figure 4:**
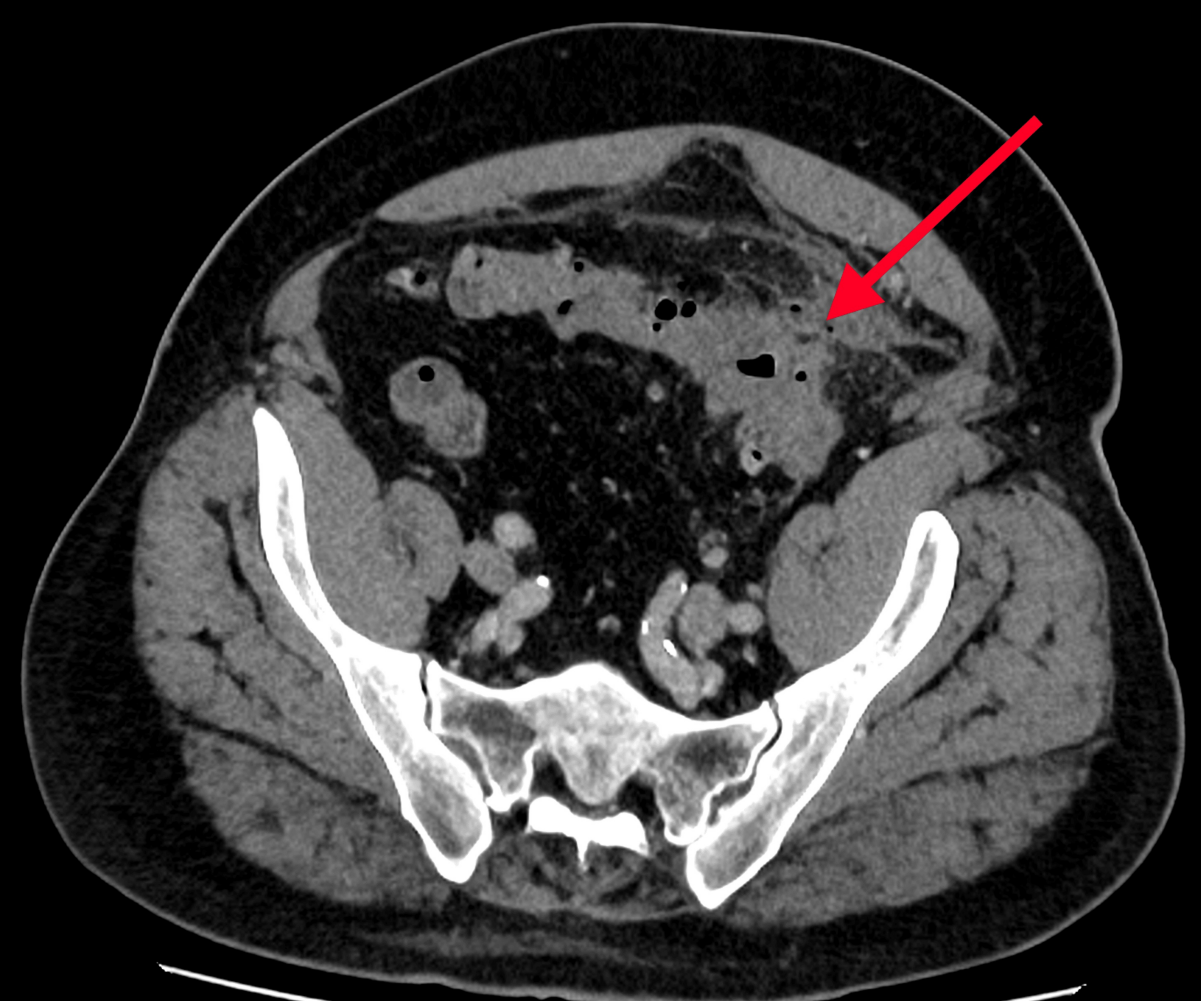
Axial view (inferior slice) of contrast-enhanced CT of the pelvis The red arrow highlights sigmoid diverticulitis with localized perforation and pericolic inflammation, representing the primary focus of disease responsible for the secondary small bowel changes.

Contrast-enhanced CT of the abdomen and pelvis (Figure 1, Figure 2, Figure 3, Figure 4) demonstrated sigmoid diverticulitis with a small localized perforation and pericolic inflammation. Adjacent small bowel loops were mildly dilated with subtle mural hyperenhancement and surrounding fat stranding, consistent with reactive change. A transition point was noted in the left iliac fossa, where the small bowel tapered adjacent to the inflamed sigmoid colon (Figure 1). The coronal and axial reconstructions (Figure 2, Figure 3, Figure 4) depict the relationship between the inflamed sigmoid colon and the adjacent reactive small bowel, suggesting an inflammatory mechanism contributing to obstruction.

The patient was managed conservatively under the emergency surgery team. He was kept nil by mouth and commenced on nasogastric decompression with four-hourly aspirates, IV fluids, and broad-spectrum IV antibiotics (cefuroxime and metronidazole) as per trust guidelines. Given the localized perforation and collection, antibiotic therapy was continued empirically for complicated diverticulitis. By day 2 of admission, the patient had not taken any oral intake for five days, and total parenteral nutrition (TPN) was initiated following dietetic assessment to prevent nutritional depletion.

On day 3, a water-soluble contrast (Gastrografin) study was performed for both diagnostic and therapeutic purposes. Follow-up abdominal radiography demonstrated contrast progression to the rectum, confirming bowel continuity and correlating with clinical improvement. Over the subsequent 48 hours, the patient began to pass flatus, his abdominal distension improved, and the nasogastric tube was removed. Oral fluids were gradually reintroduced, followed by a transition to a soft diet.

TPN was discontinued on day 5 as oral intake improved. IV antibiotics were switched to oral co-amoxiclav, completing a two-week course in total as per trust guidelines for intra-abdominal collections. The patient was discharged home on day 7 on a low-fiber diet with outpatient review arranged at two weeks. At follow-up, he reported resolution of symptoms, his blood workup showed normal inflammatory markers, and he was advised to transition to a high-fiber diet with a planned colonoscopy.

## Discussion

SBO secondary to sigmoid diverticulitis is an uncommon presentation, most often resulting from extrinsic compression by inflammatory phlegmon or abscess [3]. In this patient, the obstruction was functional rather than mechanical, arising from reactive enteric changes in small bowel loops adjacent to the inflamed sigmoid colon. The CT findings of mural hyperenhancement and mild mural edema without discrete obstruction or intrinsic small bowel pathology supported the diagnosis of reactive enteritis with functional obstruction. Recognition of this imaging pattern is crucial, as it distinguishes such cases from those requiring operative intervention [4,5].

CT played a pivotal role in guiding management by delineating the transition point, identifying the contained perforation, and confirming the absence of complete mechanical obstruction [3,5]. The imaging also demonstrated reactive enteric involvement and localized pericolic inflammation, features consistent with an inflammatory ileus rather than a fibrotic or adhesive obstruction.

Conservative management was appropriately selected for this hemodynamically stable patient. Broad-spectrum IV antibiotics (cefuroxime and metronidazole) were commenced as per trust guidelines for complicated diverticulitis with localized perforation and small collection and continued for a total of two weeks in accordance with local intra-abdominal infection guidance. Notably, while recent WSES guidelines (2020) recommend avoiding antibiotics in uncomplicated diverticulitis, their use remains indicated in complicated disease, such as in this case [6].

Nutritional support was a key component of management. As the patient had been unable to tolerate oral intake for five days, TPN was initiated to prevent catabolic deterioration. This reflected a proactive multidisciplinary approach involving the surgical and dietetic teams. The decision to commence TPN after five days of fasting was consistent with ESPEN recommendations. The ESPEN surgical guideline advises initiating parenteral nutrition when oral or enteral feeding is not feasible for more than seven days, or earlier in nutritionally at-risk patients [7].

The administration of water-soluble contrast (Gastrografin) served both diagnostic and therapeutic purposes. Radiologically, it confirmed bowel continuity and excluded a high-grade obstruction. Clinically, its hyperosmolar and motility-enhancing effects appeared to facilitate recovery in this patient, with resolution of symptoms within 48 hours. This observation is consistent with published evidence demonstrating that Gastrografin provides both diagnostic value and therapeutic benefit in selected cases of partial SBO [1].

The patient’s gradual clinical improvement, marked by the return of bowel function, tolerance of oral diet, and avoidance of surgery, highlights the success of conservative management when guided by imaging and multidisciplinary review. Recognition and targeted treatment of the primary colonic source of inflammation ultimately led to resolution of the secondary reactive enteritis, underscoring the pathophysiological link between the two processes [2,4].

## Conclusions

Reactive enteritis is a rare but clinically important mechanism of SBO occurring secondary to sigmoid diverticulitis. Recognition of this entity on CT is essential, as it can mimic mechanical obstruction yet often resolves with conservative management. In this case, controlling the primary colonic inflammation with antibiotics led to resolution of the reactive small bowel changes and restoration of normal bowel function. The adjunctive use of water-soluble contrast provided both diagnostic confirmation and a therapeutic effect, contributing to clinical improvement. Early nutritional support with TPN and adherence to local antibiotic guidelines ensured safe and effective recovery without surgical intervention. This case highlights the importance of correlating CT findings with clinical stability to guide a nonoperative approach in selected patients with complicated diverticulitis and secondary functional obstruction.
